# Supplementary material for: Multi‐dataset identification of innovative feature genes and molecular mechanisms in keratoconus
Source: J Cell Mol Med. 2024 Sep 19;28(18):e70079. doi: 10.1111/jcmm.70079 (PMC11412914; doi:10.1111/jcmm.70079)
Supplement: Supplementary file 2 — Material S2. [file JCMM-28-e70079-s002.zip › 7.GSEA analysis/3.DBNDD1/KEGG_BASAL_CELL_CARCINOMA.html]

Details for gene set KEGG\_BASAL\_CELL\_CARCINOMA[GSEA]

|  || Dataset | DBNDD1.group.cls#h\_versus\_l |
| Phenotype | group.cls#h\_versus\_l |
| Upregulated in class | l |
| GeneSet | KEGG\_BASAL\_CELL\_CARCINOMA |
| Enrichment Score (ES) | -0.4904205 |
| Normalized Enrichment Score (NES) | -1.4346849 |
| Nominal p-value | 0.06786427 |
| FDR q-value | 0.67473763 |
| FWER p-Value | 0.871 |
Table: GSEA Results Summary

  

Fig 1: Enrichment plot: KEGG\_BASAL\_CELL\_CARCINOMA      
 Profile of the Running ES Score & Positions of GeneSet Members on the Rank Ordered List

  

| SYMBOL | TITLE | RANK IN GENE LIST | RANK METRIC SCORE | RUNNING ES | CORE ENRICHMENT || 1 | WNT16 | na | 6810 | 0.129 | -0.1752 | No |
| 2 | PTCH2 | na | 8169 | 0.100 | -0.1978 | No |
| 3 | FZD5 | na | 9252 | 0.082 | -0.2156 | No |
| 4 | FZD3 | na | 9718 | 0.075 | -0.2166 | No |
| 5 | FZD4 | na | 9743 | 0.075 | -0.2049 | No |
| 6 | WNT9A | na | 9937 | 0.072 | -0.1986 | No |
| 7 | FZD1 | na | 10494 | 0.065 | -0.2040 | No |
| 8 | WNT8A | na | 10620 | 0.063 | -0.1972 | No |
| 9 | SUFU | na | 13357 | 0.035 | -0.2703 | No |
| 10 | FZD8 | na | 13456 | 0.035 | -0.2675 | No |
| 11 | TP53 | na | 13962 | 0.030 | -0.2771 | No |
| 12 | WNT10B | na | 14038 | 0.030 | -0.2743 | No |
| 13 | WNT3A | na | 14444 | 0.027 | -0.2816 | No |
| 14 | PTCH1 | na | 14497 | 0.026 | -0.2787 | No |
| 15 | FZD6 | na | 14642 | 0.025 | -0.2787 | No |
| 16 | HHIP | na | 14730 | 0.025 | -0.2772 | No |
| 17 | CTNNB1 | na | 16378 | 0.013 | -0.3225 | No |
| 18 | WNT5A | na | 17212 | 0.008 | -0.3452 | No |
| 19 | AXIN1 | na | 19524 | -0.009 | -0.4104 | No |
| 20 | TCF7L2 | na | 20073 | -0.014 | -0.4239 | No |
| 21 | DVL3 | na | 20205 | -0.015 | -0.4253 | No |
| 22 | WNT5B | na | 20321 | -0.016 | -0.4260 | No |
| 23 | APC2 | na | 21518 | -0.027 | -0.4560 | No |
| 24 | WNT4 | na | 21797 | -0.030 | -0.4591 | No |
| 25 | WNT3 | na | 21833 | -0.030 | -0.4551 | No |
| 26 | SMO | na | 22291 | -0.035 | -0.4625 | No |
| 27 | GSK3B | na | 22452 | -0.037 | -0.4611 | No |
| 28 | STK36 | na | 22907 | -0.042 | -0.4673 | Yes |
| 29 | WNT2 | na | 22923 | -0.042 | -0.4608 | Yes |
| 30 | WNT9B | na | 23327 | -0.047 | -0.4646 | Yes |
| 31 | GLI3 | na | 23514 | -0.050 | -0.4618 | Yes |
| 32 | TCF7 | na | 23571 | -0.051 | -0.4550 | Yes |
| 33 | BMP4 | na | 24168 | -0.058 | -0.4626 | Yes |
| 34 | DVL1 | na | 24203 | -0.059 | -0.4539 | Yes |
| 35 | APC | na | 24380 | -0.062 | -0.4488 | Yes |
| 36 | WNT6 | na | 24905 | -0.069 | -0.4525 | Yes |
| 37 | DVL2 | na | 25071 | -0.072 | -0.4454 | Yes |
| 38 | FZD9 | na | 25389 | -0.077 | -0.4419 | Yes |
| 39 | WNT7B | na | 26237 | -0.092 | -0.4512 | Yes |
| 40 | TCF7L1 | na | 26813 | -0.104 | -0.4507 | Yes |
| 41 | WNT10A | na | 27282 | -0.114 | -0.4453 | Yes |
| 42 | GLI2 | na | 27332 | -0.115 | -0.4277 | Yes |
| 43 | AXIN2 | na | 28854 | -0.153 | -0.4464 | Yes |
| 44 | SHH | na | 28942 | -0.155 | -0.4234 | Yes |
| 45 | WNT8B | na | 31267 | -0.231 | -0.4523 | Yes |
| 46 | BMP2 | na | 31395 | -0.236 | -0.4171 | Yes |
| 47 | FZD10 | na | 31652 | -0.246 | -0.3839 | Yes |
| 48 | WNT7A | na | 31957 | -0.261 | -0.3496 | Yes |
| 49 | FZD2 | na | 32387 | -0.287 | -0.3148 | Yes |
| 50 | LEF1 | na | 32857 | -0.317 | -0.2760 | Yes |
| 51 | WNT2B | na | 33588 | -0.378 | -0.2348 | Yes |
| 52 | GLI1 | na | 33749 | -0.397 | -0.1739 | Yes |
| 53 | WNT1 | na | 33810 | -0.404 | -0.1091 | Yes |
| 54 | FZD7 | na | 33835 | -0.408 | -0.0426 | Yes |
| 55 | WNT11 | na | 33881 | -0.415 | 0.0244 | Yes |
Table: GSEA details [plain text format]

  

Fig 2: KEGG\_BASAL\_CELL\_CARCINOMA      
 Blue-Pink O' Gram in the Space of the Analyzed GeneSet

  

Fig 3: KEGG\_BASAL\_CELL\_CARCINOMA: Random ES distribution      
 Gene set null distribution of ES for **KEGG\_BASAL\_CELL\_CARCINOMA**
